# Enhanced Compressive Mechanical Properties of Bio-Inspired Lattice Metamaterials with Taper Struts

**DOI:** 10.3390/ma18010029

**Published:** 2024-12-25

**Authors:** Shuangyin Yuan, Bingke Song, Gang Liu, Biqi Yang, Mingqiu Dai, Zetian Gao, Shan Cao, Miao Zhao

**Affiliations:** 1 Suzhou XDM 3D Printing Technology Co., Ltd., Suzhou 215000, China; 2 Shanghai Institute of Spacecraft Equipment, Shanghai 200240, China

**Keywords:** lattice metamaterials, taper struts, mechanical properties, deformation behavior

## Abstract

The stress distribution within the struts of lattice metamaterials is non-uniform under compressive loads, with stress concentrations typically occurring at the node regions. Inspired by bamboo, this study proposes a type of body-centered cubic (BCC) lattice metamaterial with tapered prism struts (BCCT). The compressive behavior, deformation modes, mechanical properties, and failure mechanisms of BCCT lattice metamaterials are systematically analyzed using finite element methods and validated through compression tests. Parametric analysis is conducted to investigate the effects of key design parameters, including volume fraction, shape parameter, and material properties. The results reveal that BCCT lattice metamaterials effectively eliminate stress concentration at nodes by redistributing stress toward the center of the struts. This redistribution changes the failure mode from shear band failure to layer collapse, while the struts maintain a bending-dominated deformation mechanism under compression. The mechanical properties of BCCT lattice metamaterials are significantly influenced by the shape factor. Furthermore, the mechanical properties of BCCT lattice metamaterials with different volume fractions and materials are consistently superior to BCC ones, which verifies the effectiveness and adaptability of lattice metamaterials with taper prismatic struts for potential lightweight applications.

## 1. Introduction

Lattice metamaterials are multifunctional materials that exhibit outstanding properties, including low weight [1], high strength [2], excellent energy absorption [3], and efficient thermal exchange [4], making them highly promising for aerospace applications. Unlike traditional foam materials, lattice metamaterials are composed of regular, pre-designed struts, allowing their performance to be tailored by modifying the internal strut configurations [5,6]. However, conventional fabrication methods have posed challenges in manufacturing lattice metamaterials with complex configurations. Recent advancements in metal additive manufacturing (AM) technologies now enable the precise and high-fidelity fabrication of lattice metamaterials with complex internal structures [7]. The performances of metamaterials manufactured via AM are influenced by geometric configurations [8] and several AM parameters [9], such as layer thickness, build orientation, and bulk materials.

The traditional approach to designing lattice metamaterials relies on computer-aided design (CAD). In this method, a single unit cell is created, and the lattice metamaterials are generated through array and union operations [10]. However, the CAD-based method is inefficient for complex designs. Recently, an implicit design method has been introduced, which represents the surfaces of lattice metamaterials using implicit surface formulas [11]. This approach enables the generation of complex lattice metamaterials through mathematical formulas, even allowing for the design of functionally graded [12], hierarchical [13], conformal [14], and heterogeneous [15] lattice metamaterials by modifying these formulas. In AM processes, the surfaces of lattice metamaterials are commonly converted into triangular facets and exported as stereolithography (STL) files for fabrication. Modeling implicit surfaces for complex geometries requires a large number of triangular facets, leading to oversized STL files that are challenging to handle in engineering applications. To address these limitations, a parametric design method for lattice metamaterials with prismatic struts has been developed [16]. This approach minimizes the number of triangular facets, significantly reducing STL file sizes, and facilitates the efficient design of large-scale lattice metamaterials [14].

The stress distribution within the struts of lattice metamaterials is non-uniform under compressive loads, with stress concentrations typically occurring at the node regions [17,18]. These stress concentrations negatively influence the mechanical properties of lattice metamaterials. To address this, researchers have explored various strategies to enhance mechanical properties, particularly by enhancing the node regions [19,20,21]. For instance, Wang et al. [22] introduced fillets at the nodes of body-centered cubic (BCC) lattice metamaterials. The fillets reduced stress concentrations at the nodes, resulting in a 55% and 24% improvement in elastic modulus and yield strength, respectively. Similarly, the BCC lattice metamaterials with minimal surfaces also have been proposed, and the smooth transition on node regions improved the mechanical properties by 2–42.2% [23]. In addition to the node optimization, modifying strut geometry offers another approach for enhancing mechanical properties [24,25,26,27]. Liu et al. [28] developed BCC lattice metamaterials with I-shaped struts, where the increased moment of inertia of the cross-section improved the mechanical properties by 41.42–159.13%. Alomar et al. [29] designed circular lattice metamaterials with stress uniformly distributed across all layers. Meanwhile, Wang et al. [30] proposed hierarchical multi-circular lattice metamaterials, which introduced additional plastic hinges and enhanced energy absorption by 20.5%. However, in the previous literature, most of the enhanced designs are based on lattice metamaterials with uniform strut cross-sections. Additionally, most of the lattice metamaterials are generated by CAD-based and implicit design methods. Limited research has investigated the effects of strut shape on the lattice metamaterials with prismatic struts generated by the parametric design method, which result in fewer triangular facets.

In nature, bamboo is a lightweight structure renowned for its exceptional strength and toughness [31]. Its trapezoidal external shape enhances bending resistance under radial loads [32]. In order to enhance the compressive mechanical properties of BCC lattice metamaterials, this study proposes a type of BCC lattice metamaterials with tapered prismatic struts inspired by the bamboo’s structural characteristic, named BCCT. The compressive behavior, deformation modes, mechanical properties, and failure mechanisms of BCCT lattice metamaterials were systematically analyzed using finite element methods (FEM) and validated through compression tests. A parametric analysis was conducted to examine the effects of key design parameters, including volume fraction, shape parameter, and material properties, on the mechanical properties of BCCT lattice metamaterials. The results demonstrate that the BCCT lattice metamaterials effectively eliminate stress concentrations at the nodes, which significantly enhances their mechanical properties. These advancements make BCCT lattice metamaterials promising candidates for aerospace applications.

## 2. Materials and Methods

### 2.1. Design of BCCT Lattice Metamaterials

**Figure 1:**
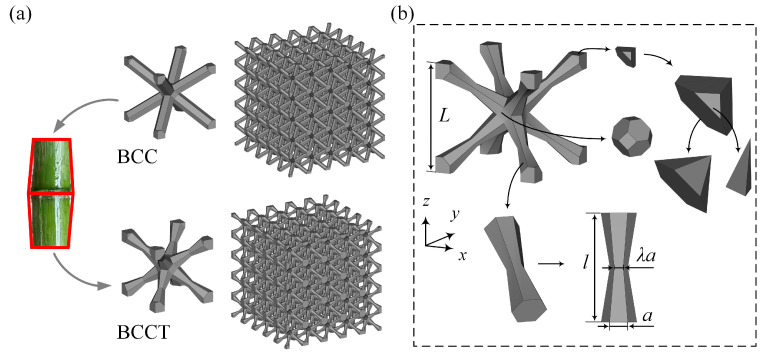
Geometry of BCC and BCCT lattice metamaterials: (**a**) CAD models of BCC and BCCT lattice metamaterials; (**b**) schematic diagram of geometry disassembly of BCCT lattice metamaterials.

Figure 1 illustrates the topological configuration of BCC lattice metamaterials, which consist of eight prismatic struts connecting the center and vertices of a cubic unit cell. To enhance the mechanical properties of BCC lattice metamaterials and reduce stress concentrations at the node regions under compressive loads, BCCT lattice metamaterials with tapered prismatic struts are proposed, as shown in Figure 1. The unit cell of the BCCT lattice metamaterials comprises eight tapered prismatic struts, with each strut consisting of two one-eighth node segments connected by a tapered beam.

In order to calculate the volume fraction (*ρ*) of the BCCT lattice metamaterials, the unit cell is divided into several simple geometries, as shown in Figure 1b. Then, the relationship between the volume of the lattice unit cell (*V*) and the design parameters can be expressed as

(1) $$V=4\sqrt{3}\left(1+\lambda +\lambda^{2}\right)a^{2}l+16\sqrt{2}a^{3},$$

where a is the length of the bottom side of the tapered prismatic struts, and *l* is the length of the tapered prismatic struts. λ is the shape factor to control the degree of taper, and the λ=1 represents the BCC lattice metamaterials. The relationship between unit cell size (*L*) and *l* is

(2) $$l=\frac{\sqrt{3}}{2}L-a.$$

Therefore, the *ρ* of BCCT lattice metamaterials can be calculated by

(3) $$\rho =\frac{V_{l}}{L^{3}}=6\left(1+\lambda +\lambda^{2}\right){\left(\frac{a}{L}\right)}^{2}-12\sqrt{2}\left(\lambda +\lambda^{2}-\frac{1}{3}\right){\left(\frac{a}{L}\right)}^{3}.$$

### 2.2. Finite Element Method

**Figure 2:**
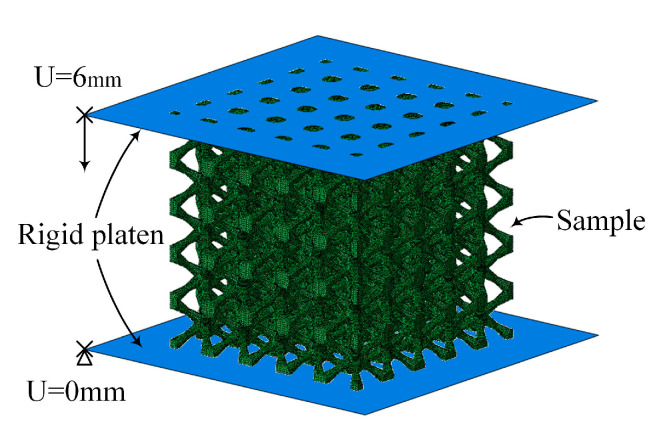
Schematic of the finite element model of BCCT lattice metamaterials.

The quasi-static compression process of the lattice metamaterials is simulated by using ABAQUS 2019. The FEM efficiently reduces the time as well as the high costs of machinery, metal powder, processing, and testing. Two rigid plates are added at the top and bottom of the lattice metamaterials to simulate the boundary conditions of the quasi-static compression process. The lower rigid plate is fixed, and the upper rigid plate moves downward, as shown in Figure 2. The friction coefficient of the contact process is 0.1. According to a previous study [33], 5 × 5 × 5 unit cells are sufficient to represent the mechanical properties of the lattice metamaterials. Therefore, the lattice metamaterials consisted of 5 × 5 × 5 unit cells, and the unit cell size was 4 mm. The lattice metamaterials and the rigid plates were meshed using C3D8R and R3D4 elements, respectively. The mesh convergency was conducted to determine the number of elements used in FEM, and each model contained 700,000 elements.

The elastic properties of the Ti-6Al-4V material used in FEM was isotropic linear elasticity with an elastic modulus of 107 GPa and Poisson’s ratio of 0.3 [34]. Since the experiments were carried out at the constant strain rate and room temperature, a simplified Johnson-Cook plasticity model was used, and the yield stress ($\sigma_{s}$) was expressed as [35]:

(4) $$\sigma_{s}=A+B{\left(\varepsilon_{e}\right)}^{N}$$

where, $\varepsilon_{e}$ is the equivalent plastic strain, and *A*, *B*, and *N* are material-related parameters. In order to simulate the damage behavior of bulk material, a simplified Johnson–Cook damage model is introduced to remove the elements after failure, and the fracture strain ($\varepsilon_{f}$) is expressed as [35]

(5) $$\varepsilon_{f}=D_{1}+D_{2}e^{\left(D_{3}\sigma^{*}\right)},$$

where $\sigma^{*}$ is the stress triaxiality, and $D_{1}$, $D_{2}$, and $D_{3}$ are material-related parameters. The parameters of the Johnson–Cook model are shown in Table 1.

### 2.3. Sample Fabrication

**Figure 3:**
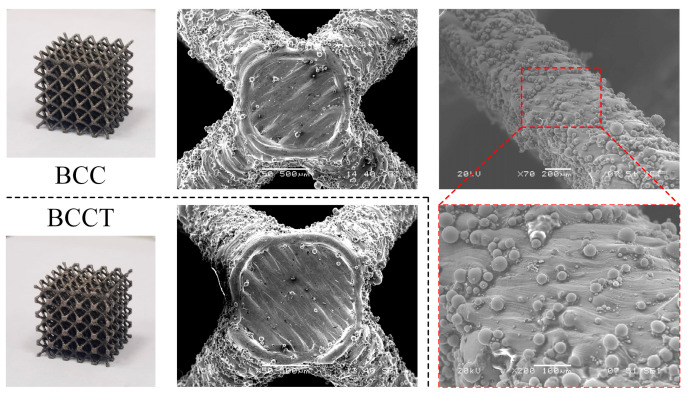
Photographs of BCC and BCCT lattice samples fabricated with LPBF.

The samples were manufactured using EOS 280 laser powder bed fusion (LPBF) equipment (EOS GmbH, Krailling, Germany), and the process parameters are shown in Table 2. To validate the FEM results, the lattice metamaterials with *ρ* = 0.13 and *λ* = 0.5 and 1 were selected for testing. Three duplications of each design were fabricated, and the fabricated samples are shown in Figure 3. All struts of the samples were well connected. It can be observed that the upper surfaces of BCC and BCCT lattice metamaterials are smooth, while there are partly melted powders adhering on the side surfaces. This is because the powders around the contour line were partially melted when the laser passed over the contour and adhered to the surface after solidification [37].

### 2.4. Measurements

The compressive characteristics of the lattice metamaterials were tested using a 100 KN universal material testing machine (Shenzhen Wance Testing Machine Co., Ltd., Shenzhen, China) with a loading speed of 2 mm/min. The bottom plate was fixed, while the top load cell moved downward. The force and displacement data were recorded and used to calculate the strain–stress curves of the lattice metamaterials. The deformation behaviors of the lattice metamaterials during the compression process were recorded using a camera.

## 3. Results and Discussion

### 3.1. Validation of FEM

**Figure 4:**
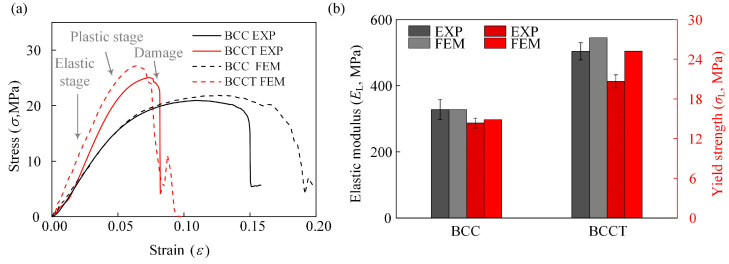
Comparison of simulation and experimental results: (**a**) strain–stress curves; (**b**) mechanical properties.

The stress–strain curves of lattice metamaterials obtained from FEM and experiments are shown in Figure 4a. The elastic modulus and yield strength of the BCC and BCCT samples were calculated based on the stress–strain curves. It can be found that the trend of the simulated and experimental curves is consistent. The comparison of the mechanical properties obtained from experiments and simulations is shown in Figure 4b and Table 3. The enhanced compressive mechanical properties of BCCT lattice metamaterials were successfully predicted. The maximum errors in elastic modulus and yield strength were 8.17% and 22.01%, respectively. The differences are mainly attributed to the ideal geometric models considered in the FEM. It is worth noting that large errors were only observed in the BCCT lattice metamaterials, whereas the maximum error of the BCC lattice metamaterials was 3.48%. The large errors observed in BCCT lattice metamaterials might be due to the tapered design, which reduced the feature size at the center of the struts. These small features are challenging to precisely fabricate with LPBF, resulting in reduced mechanical properties of the BCCT struts.

**Figure 5:**
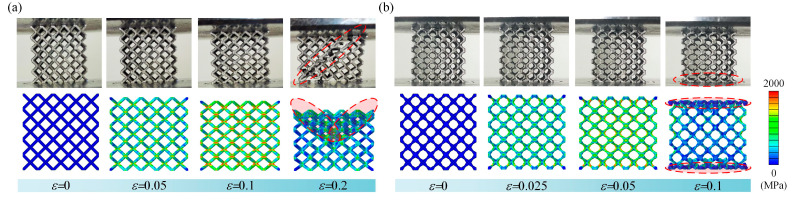
Deformation behaviors of lattice metamaterials obtained using FEM and experiments: (**a**) BCC; (**b**) BCCT.

Figure 5 illustrates the distribution of von Mises stress in BCC and BCCT lattice metamaterials during the compression. For BCC lattice metamaterials, the von Mises stress was mainly concentrated in the node region. As the strain increased, a shear failure was observed, which is similar to the failure behavior observed in the experiment. In contrast, for BCCT lattice metamaterials, the von Mises stress was transferred to the central regions of the struts. As the strain increased, the top and bottom layers simultaneously collapsed, consistent with the layer failure observed in the experiment. The results show that the simulation results are in good agreement with the experiments, validating the effectiveness of FEM in predicting the compressive responses of both BCC and BCCT lattice metamaterials.

### 3.2. Deformation Behaviors

The stress–strain curves of the lattice metamaterials obtained from the compression tests are presented in Figure 4a. These curves can be divided into three distinct stages: elastic, plastic, and damage. During the elastic stage, the BCCT lattice metamaterials exhibited a steeper slope compared to the BCC ones, indicating that the tapered strut design enhanced the stiffness of the lattice metamaterials. However, in the plastic stage, the BCC lattice metamaterials demonstrated a longer plateau stress, suggesting that the tapered strut design reduced toughness. This reduction in toughness is attributed to the changes in the failure mode of the lattice metamaterials, as illustrated in Figure 5. Under compressive deformation, the BCC lattice metamaterials underwent shear band failure across the whole structure. In contrast, the BCCT lattice metamaterials collapsed in the bottom layer. Furthermore, the von Mises stress distribution differed between the two designs. As shown in Figure 5a, for the BCC lattice metamaterials, the von Mises stress concentrated at the nodes and extended along diagonal regions, enabling all layers to contribute to resisting compressive loads. This requires a larger compressive strain for the complete failure, which enhanced toughness of the BCC lattice metamaterials. Conversely, for the BCCT lattice metamaterials, the von Mises stress was primarily concentrated in the top and bottom layers, leading to earlier failure and reduced toughness, as shown in Figure 5b. When initial damage occurred, the stress–strain curves showed a significant drop, accompanied by a substantial reduction in the load-bearing capacity of both the BCC and BCCT lattice metamaterials.

**Figure 6:**
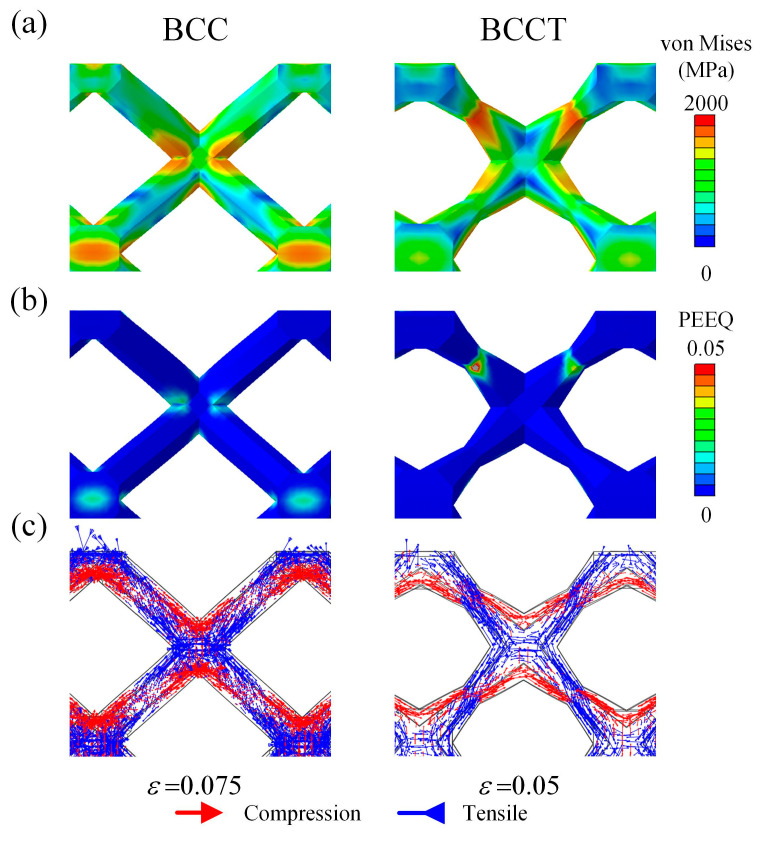
FEM results of BCC and BCCT unit cells: (**a**) von Mises stress distribution; (**b**) plastic strain distribution; (**c**) principal stress distribution.

To investigate the deformation mechanism of the lattice metamaterials, the unit cells in the top layer in the plastic stage were analyzed. The von Mises stress distribution and principal stress distribution are presented in Figure 6. For BCC unit cells, the von Mises stress was concentrated in the node regions. Tensile stress is predominantly distributed on the upper and lower regions of the nodes, while compressive stress was mainly localized on the left and right sides. According to the previous studies, the failure of the struts was primarily attributed to the combined effects of tensile stress and stress concentration [18]. Thus, the fractures of BCC lattice metamaterials occurred in the node regions. However, for BCCT unit cells, both von Mises stress and tensile stress shifted toward the center of the struts. This redistribution caused the failure location of the BCCT lattice metamaterials to move from the node regions to the center of the struts, as shown in Figure 5. Notably, the tensile and compressive stress distributions in both BCC and BCCT lattice metamaterials were symmetrical along the struts, which is a typical bending deformation mode. These findings indicate that the design of tapered struts has a little effect on the deformation mode of the lattice metamaterials under compressive loads.

**Figure 7:**
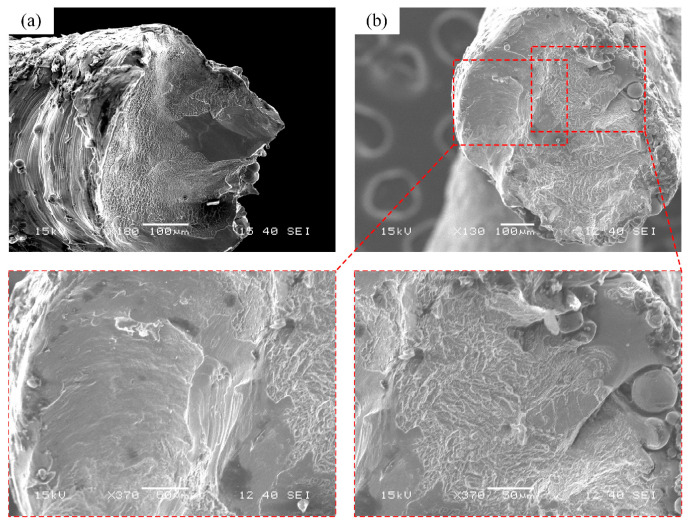
Fracture surfaces of lattice metamaterials after compression tests: (**a**) BCC; (**b**) BCCT.

Figure 7 shows the fracture surfaces of the BCC and BCCT samples after compression tests. There were two distinct patterns: deep ductile dimples and smooth cleavage features. Cracks typically initiate at adhered powder particles or microcracks due to the combined influence of stress concentration and high tensile stress. During the compression process, tensile stress increased, and the cracks rapidly propagated into the interior of the struts [38], resulting in smooth cleavage fracture planes. As the stiffness of the lattice structure reduced, the struts were torn and produced numerous deep ductile dimples. Therefore, the fracture mechanisms of both BCC and BCCT lattice metamaterials involve a combination of brittle and ductile failure modes.

### 3.3. Mechanical Properties

**Figure 8:**
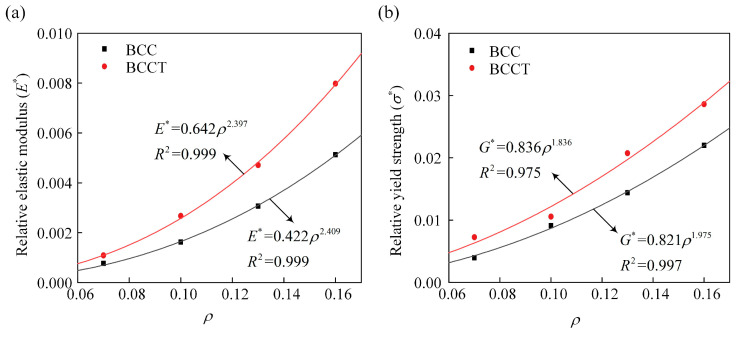
Relationships between relative mechanical properties and volume fraction: (**a**) elastic modulus; (**b**) yield strength.

The comparison of the elastic modulus and yield strength between the BCC and BCCT lattice metamaterials is shown in Table 3. The experimental results show that the BCCT lattice metamaterials had higher mechanical properties, with increases of 53.6% in elastic modulus and 43.9% in yield strength compared to their BCC ones. Typically, the mechanical properties of lattice metamaterials are closely related to their deformation mechanisms. Based on the deformation mode of struts under compressive loading, lattice metamaterials are classified as stretching-dominated and bending-dominated. The stretching-dominated lattice metamaterials exhibit superior mechanical performance. To reveal the deformation mechanism of BCCT lattice metamaterials, the Gibson-Ashby model was employed to fit the relationship between mechanical properties and volume fraction. The mechanical properties of BCC and BCCT lattice metamaterials with different volume fractions were simulated by FEM, and the fitted Gibson–Ashby models are shown in Figure 8. Previous studies indicate that the Gibson–Ashby exponent is ~2 for bending-dominated and ~1 for stretching-dominated lattice metamaterials [39]. In this study, the design of taper strut had little influence on the exponent, with all values remaining ~2. This confirms that both BCC and BCCT lattice metamaterials exhibit bending-dominated deformation mechanisms under compressive loads. Therefore, the improved mechanical properties of the BCCT lattice metamaterials were not attributed to a shift in deformation mechanisms.

**Figure 9:**
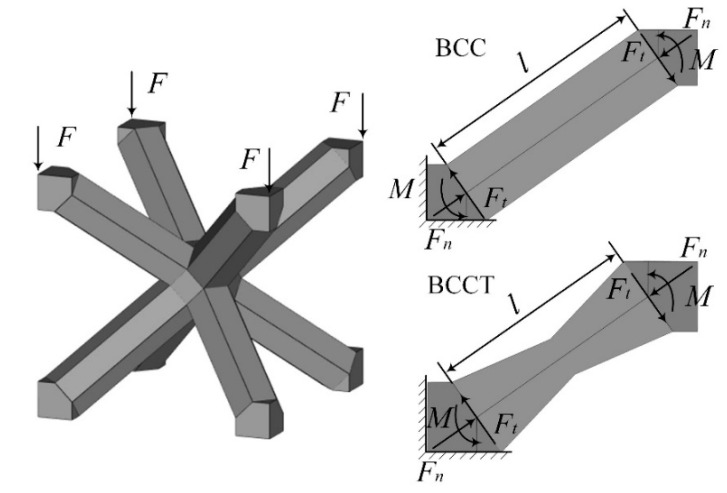
Strut of BCC and BCCT lattice metamaterials with boundary conditions.

Two factors contribute to the enhanced mechanical properties of BCCT lattice metamaterials. First, the tapered strut design reduced stress concentrations at the nodes and shifted the failure region to the center of the struts. This more uniform stress distribution enhanced the mechanical properties of the lattice metamaterials. Second, due to the bending-dominated deformation mode, the struts primarily underwent bending deformation under compressive loads. The geometries of the struts with boundary conditions are demonstrated in Figure 9. One end of each strut is fixed, while the other end is free. The applied compressive force is decomposed into an axial force ($F_{n}$) and a radial force ($F_{t}$). Then, the bending moment (M) of struts can be expressed as

(6) $$M=F_{t}l,$$

where *l* is the length of struts. The design of taper struts increased the volume of node regions and reduced the *l*, which further reduced the M and improved the mechanical properties of the BCCT lattice metamaterials.

### 3.4. Parametric Study

In this section, the effects of design parameters, *ρ*, *λ*, and bulk materials on the compressive responses of BCCT lattice metamaterials will be investigated.

#### 3.4.1. Effects of ρ

**Figure 10:**
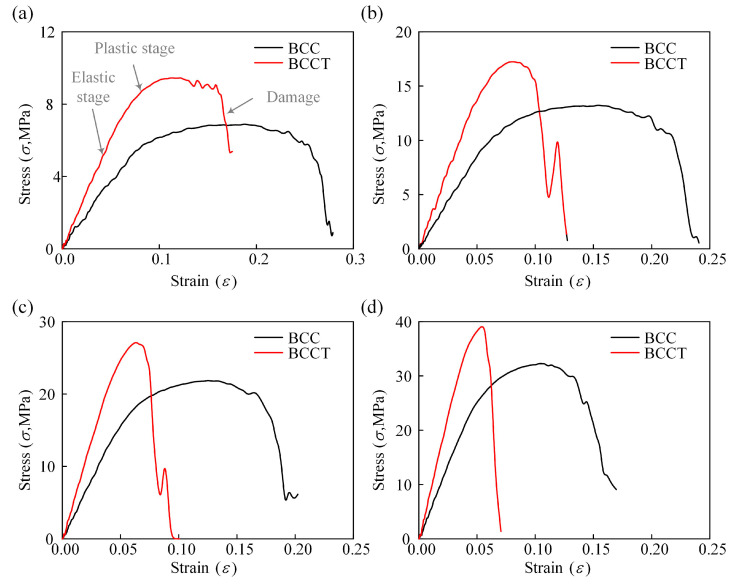
Strain–stress curves of BCC an BCCT lattice metamaterials with different *ρ*: (**a**) *ρ* = 0.07; (**b**) *ρ* = 0.1; (**c**) *ρ* = 0.13; (**d**) *ρ* = 0.16.

**Figure 11:**
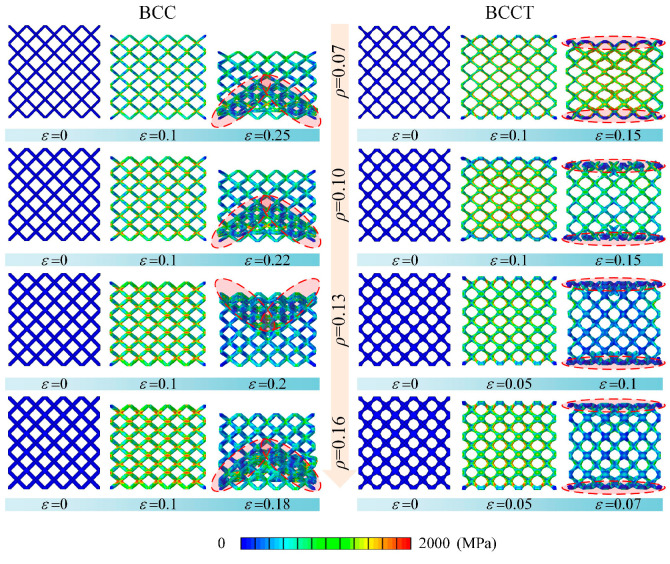
Deformation behaviors of BCC an BCCT lattice metamaterials with different *ρ* during compression process.

The compressive responses of BCCT lattice metamaterials with *ρ* = 0.07, 0.1, 0.13, and 0.16 were simulated using FEM, as shown in Figure 10 and Figure 11. For all cases, the stress–strain curves exhibited three distinct stages: elastic, plastic, and damage. During the elastic stage, the stress in BCC lattice metamaterials was concentrated at the node regions, whereas in BCCT lattice metamaterials, stress shifted toward the center of the struts. After the peak stress, BCC lattice metamaterials experienced a shear band failure, while BCCT lattice metamaterials collapsed in the top and bottom layers. The failure modes of both structures remained unaffected by the change in volume fraction.

**Figure 12:**
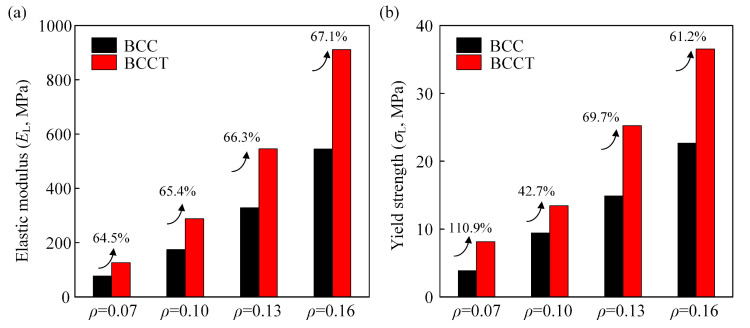
The effects of *ρ* on mechanical properties of BCC and BCCT lattice metamaterials: (**a**) elastic modulus; (**b**) yield strength.

Figure 12 illustrates the elastic modulus and yield strength of BCC and BCCT lattice metamaterials at various volume fractions. For all *ρ* values, BCCT lattice metamaterials outperformed BCC ones, with elastic modulus and yield strength improvements ranging from 64.5 to 67.1% and from 42.7 to 110.9%, respectively.

#### 3.4.2. Effects of λ

**Figure 13:**
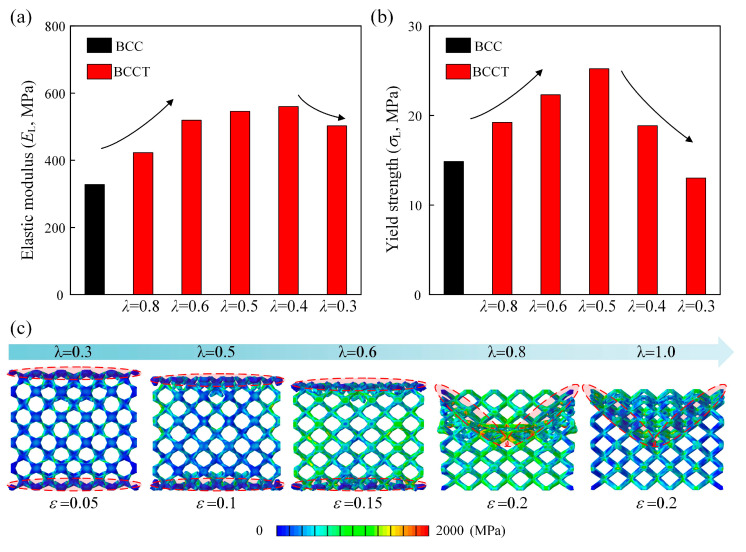
The effects of *λ* on mechanical properties and failure mode of BCCT lattice metamaterials: (**a**) elastic modulus; (**b**) yield strength; (**c**) failure mode.

The mechanical properties of BCCT lattice metamaterials with *ρ* = 0.13 and *λ* = 0.3, 0.4, 0.5, 0.6, and 0.8 were simulated using FEM, as shown in Figure 13. The results reveal that, as *λ* decreased, both the elastic modulus and yield strength initially increased and then decreased, indicating that an optimal *λ* can maximize mechanical properties. The BCCT lattice metamaterials achieved their highest elastic modulus at *λ* = 0.4, while the maximum yield strength occurred at *λ* = 0.5. The maximum improvement in elastic modulus and yield strength was 70.7% and 69.7%, respectively. For smaller *λ* values, the yield strength became more sensitive to the change of *λ*. When *λ* increased to 0.3, the elastic modulus only increased by 53.3%, while the yield strength decreased by 12.5%. Interestingly, the failure mode of BCCT lattice metamaterials can be controlled by the *λ*, and the failure mode transitioned from the shear band failure to the layer collapse when *λ* decreased, as shown in Figure 13c.

**Figure 14:**
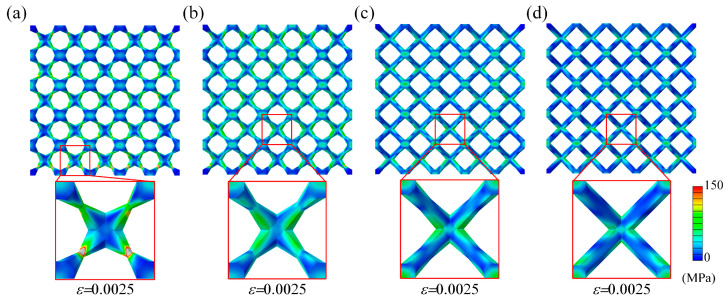
Von Mises stress distribution of BCCT lattice samples with different *λ*: (**a**) *λ =* 0.3; (**b**) *λ =* 0.5; (**c**) *λ =* 0.8; (**d**) *λ =* 1.

Figure 14 illustrates the von Mises stress distribution of BCCT lattice metamaterials for various *λ* values. As *λ* decreased, stress was redistributed from the nodes to the strut centers. At *λ* = 0.3, the stress concentrated in the central area of the struts, which in turn led to a decrease in the mechanical properties of BCCT lattice metamaterials. The results indicate that the stress concentration at nodes can be eliminated by adjusting the design parameter *λ*, which is beneficial for obtaining lightweight lattice metamaterials with excellent mechanical properties.

#### 3.4.3. Effects of Materials

**Figure 15:**
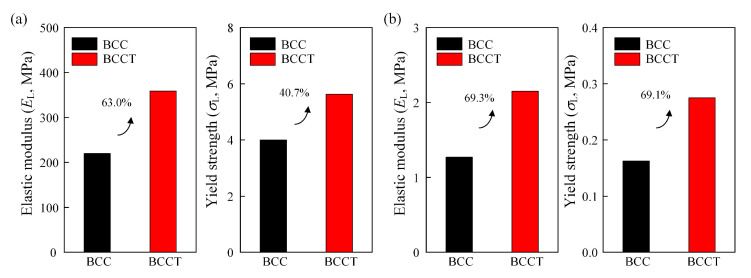
The effects of materials on mechanical properties of BCCT lattice metamaterials: (**a**) AlSi10Mg; (**b**) tough resin.

The mechanical properties of BCCT lattice metamaterials made of different materials were analyzed using FEM, as shown in Figure 15. The used material properties of AlSi10Mg and tough resin for FEM were obtained from previous studies [40,41]. For all cases, the BCCT lattice metamaterials exhibited superior performance compared to the BCC ones. For AlSi10Mg, elastic modulus and yield strength increased by 63.0% and 40.7%, respectively, while for tough resin, the increases were 69.3% and 69.1%. These results validate the versatility of the tapered strut design across various materials, highlighting the potential of BCCT lattice metamaterials for lightweight applications.

## 4. Conclusions

This study proposed novel BCCT lattice metamaterials with tapered prismatic struts inspired by the features of bamboo. The BCCT lattice metamaterials were fabricated using LPBF and were compared with traditional BCC ones. The deformation behavior, mechanical properties, and failure mechanisms were analyzed using compression tests and FEM. Finally, parametric analysis was applied to explore the effects of design parameters on the mechanical properties of BCCT lattice metamaterials. The main conclusions are as follows:

(1) BCCT lattice metamaterials eliminated stress concentration at nodes by redistributing stress to the center of the struts, resulting in a change in failure mode from shear band failure to layer collapse. Both BCC and BCCT lattice metamaterials exhibited a combination of brittle and ductile failure mechanisms.
(2) Both BCC and BCCT lattice metamaterials underwent bending deformation during compression, with tensile and compressive stresses distributed symmetrically along the struts. According to the Gibson–Ashby model, the deformation mechanism of both structures was bending-dominated, and the tapered strut design minimally influenced the deformation mode of the lattice metamaterials.
(3) The experimental results show that, compared to BCC lattice metamaterials, the elastic modulus and yield strength of the BCCT ones increased by 53.6% and 43.9%, respectively. The improved mechanical properties were attributed to the design of taper struts, which ensured more uniform stress distribution and reduced the bending moment.
(4) The mechanical properties of BCCT lattice metamaterials were controlled by the shape factor *λ*. For BCCT lattice metamaterials made of Ti-6Al-4V with *ρ* = 0.13, the highest elastic modulus and yield strength was achieved at *λ =* 0.4 and *λ =* 0.5, respectively. Compared to BCC lattice metamaterials, the improvements in elastic modulus and yield strength were 70.7% and 69.7%, respectively.
(5) The mechanical properties of BCCT lattice metamaterials with different volume fractions and materials were consistently superior to the BCC ones.

The proposed design strategy improved the compressive mechanical properties of BCC lattice metamaterials, demonstrating potential for lightweight applications. However, this study focused on the quasi-static compressive performances of BCCT lattice metamaterials. In practical engineering applications, lattice metamaterials can also serve as effective materials for absorbing impact loads. In the future, the dynamic mechanical properties of BCCT lattice metamaterials will be explored using FEM and drop-weight impact tests. In addition, this study only investigated several design parameters, and future work will investigate more design parameters, such as unit cell size.

## Figures and Tables

**Table 1:** The constants of Johnson–Cook plastic and damage models [34,36].

A (MPa)	B (MPa)	N	$D_{1}$	$D_{2}$	$D_{3}$
1567	952	0.4	−0.09	0.25	−0.5

**Table 2:** Processing parameters for LPBF.

Laser Powder (W)	Hach Space (mm)	Layer Thickness (µm)	Scan Speed (mm/s)	Oxygen
175	0.1	30	1250	<0.1%

**Table 3:** Comparison of mechanical properties of BCC and BCCT lattice metamaterials obtained by FEM and experiments.

Sample	Elastic Modulus (*E*_L_, MPa)		Error (%)	Yield Strength (*σ*_L_, MPa)		Error (%)
	Experiment	Simulation		Experiment	Simulation	
BCC	328.16 ± 30.18	327.82	−0.10	14.36 ± 0.75	14.86	3.48
BCCT	504.08 ± 25.87	545.28	8.17	20.67 ± 0.98	25.22	22.01

## Data Availability

The datasets presented in this article are not readily available due to technical/time limitations. Requests to access the datasets should be directed to the corresponding author.
